# Craniofacial Development Is Fine-Tuned by Sox2

**DOI:** 10.3390/genes14020380

**Published:** 2023-01-31

**Authors:** Nikolaos Panagiotis Mandalos, Aikaterini Dimou, Maria Angeliki Gavala, Efstathia Lambraki, Eumorphia Remboutsika

**Affiliations:** 1 University Research Institute of Maternal and Child Health & Precision Medicine, School of Medicine, National and Kapoditrian University of Athens, 115 27 Athens, Greece; 2 National Cancer Institute, Frederick, MD 21702, USA; 3 Center for Translational Medicine and the Lewis Katz School of Medicine, Temple University, Philadelphia, PA 19140, USA; 4 National Technical University of Athens, 157 80 Athens, Greece; 5 Polytechnic School, Aristotle University of Thessaloniki, 541 24 Thessaloniki, Greece; 6 Thrivus Institute for Biomedical Science and Technology, Constellations Ave, Accra GT-336-4330, Ghana

**Keywords:** neural crest stem cells, neural stem cells, embryonic stem cells, induced pluripotent stem cells, neural progenitor cells, neurogenesis, development, differentiation, regeneration, Sox1, Sox3, Sox14, Sox21, SoxB1, SoxB2, SoxB genes

## Abstract

The precise control of neural crest stem cell delamination, migration and differentiation ensures proper craniofacial and head development. Sox2 shapes the ontogeny of the cranial neural crest to ensure precision of the cell flow in the developing head. Here, we review how Sox2 orchestrates signals that control these complex developmental processes.

Cranial neural crest cells (CNCCs) are a cell population that contributes to the formation of the vertebrate head during embryogenesis [1,2,3]. They are generated from differentiated multipotent neural crest cells (NCCs), which are believed to retain their multipotency until they differentiate into craniofacial tissue progenitors. One of the main developmental processes that regulate the differentiation of CNCCs is the migration of NCCs, which delaminate, undergoing epithelial-to-mesenchymal transition (EMT), from the dorsal neural tube at E8.5 in mice [4,5,6,7]. The process of CNCC differentiation is completed by transition of the cranial mesenchyme to the epithelium. This leads to the formation of the cartilage, bone, cranial neurons, glia and connective tissue of the face. Disruption of these crucial developmental processes leads to the generation of congenital birth defects called neurocristopathies [8,9].

During development, Sox2 entertains multiple roles in pluripotency and multipotency in cellular programming and reprogramming. In the early stages of development, Sox2 preserves the pluripotent identity, while later and throughout life, Sox2 safeguards the multipotent identity. The Sox2 protein plays a pivotal role in the establishment, survival and maintenance of pluripotent cells, embryonic stem cells (ES), epiblast stem cells (EpiSC) and epiblasts, as well as in the reprogramming process that induces somatic cells to adopt a pluripotent state in induced pluripotent stem cells (iPS) [10,11,12]. Later, it is essential for maintaining the stem and progenitor pool, which is essential to establishing and maintaining a number of embryonic lineages, as well as for the maintenance in post-natal life [10,13,14,15,16,17,18].

When it comes to neuroepithelial cells and neural stem cells, which are present in the neurogenic niches of embryonic and adult brains, Sox2 plays an integral part in maintaining stem cell identity and potency before it is downregulated, which allows the stem cells to proceed with their differentiation program. Despite the fact that SoxB1 factors Sox1–3 are well established as having a role in determining the neuronal progenitor identity and suppressing neurogenesis or astrogenesis, as well as maintaining selected neuronal populations, they also play indirect and redundant roles in oligodendrocyte differentiation [13,19,20,21]. Given that both SoxB1 and Sox21 factors are expressed in the proliferating zones in the CNS, harboring antagonistic effects, they might determine if a cell will undergo self-renewal or undergo cell cycle exit, depending on their relative activity [13,22,23,24,25]. 

Sox2 acts in a cell-autonomous manner during NCC development [15,16,22,23]. The induction of NCCs is accompanied by neural plate induction, a process that could not possibly exclude Sox2, an early neural plate marker [21,25,26,27]. Sox2 is upregulated in the ectoderm overlying the primitive streak [21,22,23]. Sox2 is present in the surface ectoderm region adjacent to the hindbrain in the ectoderm overlying the first branchial arch, while it is downregulated in the r5 and r6 rhombomeres [21]. Surface ectoderm Sox2 expression is restricted to the second branchial arch and the ventral half of the otic and nasal placodes [21,22,23]. Later, Sox2 is expressed ventrally in r5 in a complementary manner to Sox1 [12,13,14,15,21,22]. The neural plate expression of Sox2 is downregulated as the NCC segregate and migrate from the dorsal neural tube [21,28], while SoxE activities become strongly induced when neural stem cells transform into NCCs [22]. Elevated Sox2 levels in the embryonic ectoderm and neural plate explants inhibit NCC formation in chick and mouse embryos [15,16,22,23,28], whereas, later in migratory and post-migratory NCCs in the PNS, de novo Sox2 expression marks the onset of proliferation and subsequent differentiation events [29,30,31]. NCC development requires a switch from SoxB to SoxE gene action, with Sox2 inactivated in newly migrating trunk NCCs, whereas Sox8 is active during the induction and delamination of NCCs, while Sox9 and Sox10 are activated in newly migrating trunk NCCs [13,15,22,23,24,28].

During development, Sox2 controls the clock that allows the precise timing and aversion of genetic heterochrony events in such a way that regulates the dynamics of the decisions to generate neural crest stem cells from neural progenitors [15,16,22]. An epiblast-specific gene ablation revealed a novel specific threshold-dependent requirement for Sox2 during the epithelial-to-mesenchymal transition, leading to mammalian NCC development in vivo [13,15,22]. The downregulation of Sox2 in the epiblast leads to an abnormal accumulation of Sox10^+^ NCCs in the branchial arches and the frontonasal process. Mutant embryos exhibit severe anterior malformations, hydrocephaly and frontonasal truncations. Elevated Sox10 levels and high numbers of Sox10+-migrating NCCs in the Sox2 epiblast-deficient embryos revealed that Sox2 controls the flow of the epithelial-to-mesenchymal transition, leading to NCC delamination and migration at appropriate regions at appropriate numbers in the head region [13,15,22]. The Sox2-interacting chromatin remodeling helicase (Chd7) complex controls the formation of craniofacial structures, while the failure of this complex formation provides a conceivable explanation for several malformations associated with CHARGE syndrome, a well-studied type of neurocristopathy. Sox2, along with other pluripotent transcription factors such as Oct4 and Nanog, activate a downstream expression of FoxD3 required for the differentiation of NCCs [26,27]. 

Craniofacial abnormalities are observed with the exposure of early neural plate-stage embryos (E8.0–E8.5) to excessive retinoid acid (RA) [27,29]. During the initial stages of craniofacial development, morphological changes occur as a result of the segment-specific pattern of gene expression in the early hindbrain and its adjacent neural crest cell niche [29]. As a result, CNCCs could be the primary target of RA-induced morphological changes of craniofacial structures. This notion stems from the observation that most dysmorphic structures in RA deficiency are CNCC derivatives, suggesting that excess RA could alter CNCC survival and migration [29]. The interplay between Sox2 and RA signaling in craniofacial development remains elusive.

The Hippo signaling pathway, the main orchestrator of organ size in animals, acts as a regulator of cell proliferation, stem cell self-renewal and apoptosis (Figure 1). In this way, the Hippo signaling pathway regulates cellular and immune homeostasis, regeneration and repair and cancer development. The pathway comprises a kinase cascade, acting mainly on the YAP/TAZ transcriptional cofactors, the TEAD/TEF family transcription factors [30]. The depletion of YAP in mice leads to embryonic lethality at the gastrulation stage when body axis elongation occurs (E8.5). Although the body axis of these mutants is shorter, the arrest of development appears to be due to the disruption of the yolk sac vascularization, while the vasculogenesis of the embryo seems intact [31]. On the other hand, the suppression of YAP homolog expression, TAZ, in Taz-null/lacz mice leads to much later stage embryonic lethality (E15.5), pointing to TAZ regulating proper kidney and lung organogenesis [32]. 

During early mammalian development, Sox2 antagonizes the Hippo pathway by directly repressing two Hippo activators, restricting Sox2 expression to the inner cell mass (ICM) to regulate growth via cell proliferation and apoptosis [33] (Figure 2). Later in development, the role of the Hippo signaling pathway in CNCC development is poorly understood. Other signaling pathways, including Wnt, Fgf, Bmp and Notch, have been shown to play important roles in regulating neural crest induction, proliferation and migration [34,35,36,37,38]. Sox2 and Sox3 expression can be driven by FGF signaling, so a balance of FGF, BMP and WNT control EMT and MET during the formation of the primitive streak and the head compartments in gastrulation [39,40]. There is evidence demonstrating that the structure and the functional integrity of the extracellular matrix regulates the function of most of these factors [41]. 

In embryonic stem cells (ESCs), the nucleation of Yap enhances the binding of Nanog, Sox2 and Oct4 at YAP-binding loci [42]. Yap^-/-^ ESCs retain their undifferentiated Sox2^+^ Oct4^+^ embryonic stem cell characteristics, while Yap overexpression forces ESC to exit the cell cycle, driving lineage-specific differentiation. Thus, Yap1 levels orchestrate the balance between pluripotency and multipotency in ESCs [43], while reprogramming to induced Pluripotent stem Cells (iPSCs) can be achieved by Oct3/4, Sox2 and Yap overexpression [44,45] (Figure 2). 

In mammals, the first cellular differentiation event is the segregation of the trophectoderm and the inner cell mass (ICM). Ectopic nuclear overexpression of YAP1 in the ICM is sufficient to repress Sox2 expression, an event required for the induction of trophectoderm cell fate [46]. Later on, the protein complex YAP/Tead cooperates with TGFβ-induced signals to activate Sox2 expression during the determination of the airway epithelium cell fate [47]. Sox2 activates YAP1, influencing self-renewal and lineage fate determination during the differentiation of mesenchymal stem cells to osteogenic or adipocytic fates [48]. The YAP/Tead complex maintains the progenitor cell self-renewal in the organs with Corti progenitor cells, while Sox2 establishes the progenitor pool and initiates their hair cells, implying, in part, an antagonist role of Sox2 and the Yap/Tead complex [49]. 

Sox2 and Hippo signaling interplay has been widely studied in carcinogenesis and metastasis (Figure 2). The transcriptional activation of Sox2 by TAZ is essential for the maintenance of the stemness in head neck squamous cell carcinoma [50]. The regulation of YAP1 through Sox2 could be indirect in squamous cell carcinoma, as Sox2 antagonizes WWC1 to drive YAP1 activation [51]. In osteosarcoma stem cells, the abolishment of Sox2 expression has a similar effect on cell proliferation arrest as the abolishment of YAP, a fact that comes as no surprise, since YAP is a direct target of Sox2 in osteoblasts [52]. Sox2 antagonizes the Hippo pathway by repressing two Hippo activators, namely Nf2 and WWC1, resulting in an exaggerated YAP function marking the cancer stem cell fraction of the tumor. The differentiated fraction has high levels of Nf2 and WWC1 and reduced YAP expression in osteosarcomas, a mechanism conserved also in glioblastomas [53]. A synergistic action of Sox2 and TAZ has been reported in hepatocellular carcinoma (HCC) not only to promote cell proliferation but also to promote the epithelial-to-mesenchymal transition (EMT) for the initiation of metastatic invasion [54]. In pancreatic cancer, MBD3 regulates pancreatic cancer stem cell stemness, interacting with YAP, suppressing YAP nuclear translocation and thus suppressing both Hippo signaling and Sox2 expression [55]. A recent work revealed the synergistic role of Sox2 and the LATZ/YAP/TAZ signaling cascade in pituitary stem cells, essential for the regulation of pituitary gland size during embryogenesis and adulthood, while any dysregulation that sustains the expression of YAP alone leads to tumorigenesis by increasing the Sox2 levels in pituitary stem cells [56]. 

Neural crest cells (NCCs) were long thought to behave as multipotent stem cells that give rise to mesodermal and ectodermal lineages, including osteoblasts, chondrocytes, smooth muscle cells, neurons, glia and melanocytes, up until recently, when their pluripotent dynamics were revealed [23]. They stem from the dorsal neural tube during gastrulation, so as to contribute to the formation of multiple tissues and organs in the embryo, including the craniofacial structures. As Hippo signaling is conserved evolutionally and functionally, it is no surprise that it plays a central role in NCC development. Hippo signaling regulates Pax3 activity during the generation of NCCs at the dorsal neural tube, as TAZ/YAP65 act as coactivators of Pax3 in a Tead-independent manner in pre-migratory NCCs. Additionally, Pax3 and Hippo signaling synergistically coordinate melanocyte gene expression in the neural crest [57]. The well-conserved Notch signaling and Hippo signaling pathways converge to promote the vascularization of craniofacial structures in a mechanism that involves direct Yap–Notch intracellular domain interactions and the activation of Notch downstream target genes in a Tead-independent manner [36] (Figure 2). 

A pivotal role of Hippo signaling is reported in human NCCs, in which it synergistically acts with retinoid acid for the generation, migration and differentiation of NCCs from human pluripotent stem cell lines [58,59]. The Yap/Taz signaling complex affects the formation of craniofacial structures, including the calvaria, mandible and most midfacial bones. In addition, it contributes to the formation of the teeth, as Yap is expressed in dental epithelia and mesenchyma, while its overexpression leads to teeth malformation [60]. The genetic analysis of NCCs in zebrafish has revealed that both Yap and Sox10 are expressed in melanocytes and that their ablation affects proper NCC migration and differentiation [61]. These data suggest that SoxE genes act under the same genetic pathway as Hippo signaling in NCC development. Wnt and BMP signaling stimulate the EMT of trunk NCC emigration (EMT) through Hippo signaling in a YAP-dependent manner, maintaining proliferation in a timely fashion to Sox2 expression [15,62]. Notch signaling has not been yet shown to be necessary for NCC EMT, but it establishes the roof plate from which NCCs emigrate [58]. BMP, TGFβ, Wnt, FGF, Hippo and Notch are instrumental to NCC development and metastasis. The deregulation of these signaling pathways promotes the generation of cancer metastasis as a conservation of EMT, a process common to NCC migration and metastasis [63]. CNCCs, a subclass of NCCs, contribute to the formation of the frontal craniofacial tissues. In this developmental process, a series of signaling pathways, including BMP, Wnt1, FGF, Notch, Hedgehog and PDGF signaling, orchestrate the tissue lineage specification of CNCCs [64,65]. 

The conditional deletion of Yap/Taz in CNCCs, using Wnt1(Cre), gives a drastic phenotype, including hydrocephaly and hemorrhage in the branchial arches. It is worth mentioning that a similar phenotype has been observed in the mosaic conditional deletion of Sox2 in the epiblast using Sox2(Cre) [15,32]. We propose that Sox2 interacts with the Hippo signaling in a feedback loop regulation mode during craniofacial development (Figure 2). Future studies will demonstrate whether Sox2 interacts with Hippo signaling in an agonistic or antagonistic manner during the delamination, EMT, migration and differentiation of CNCCs. 

## Figures and Tables

**Figure 1:**
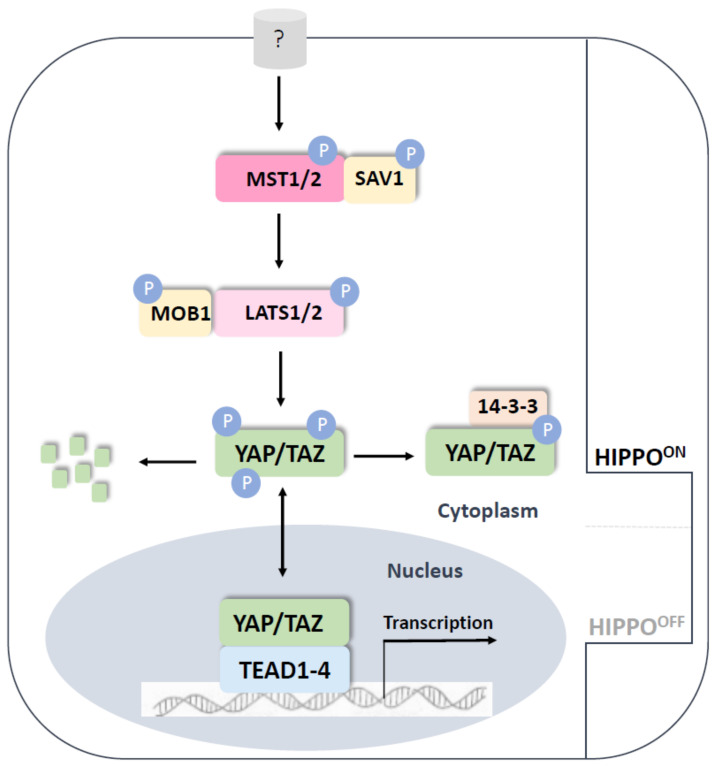
The Hippo pathway in mammals. The core Hippo pathway in mammals is composed of Mst1/2, Sav1 and Lats1/2 kinases. When Hippo signaling is ON, this kinase cascade is sequentially phosphorylated, eventually resulting in the phosphorylation of Yap and Taz to promote their interaction with the 15-3-3 protein, leading to their cytoplasmic degradation. When Hippo signaling is OFF, Yap and Taz shuttle into the nucleus and associate with other transcription factors (i.e., Tead), so as to regulate the expression of target genes involved in cell proliferation, differentiation and migration.

**Figure 2:**
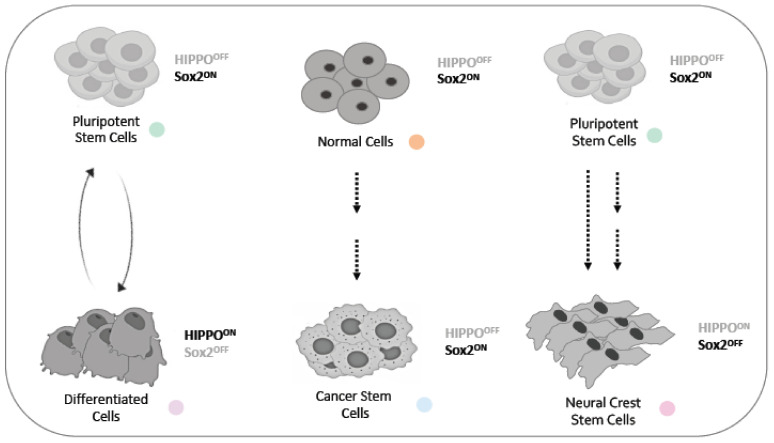
A model for the Hippo pathway and Sox2 crosstalk in healthy and diseased cells.

## Data Availability

Not applicable.
